# Risk factors for distal symmetric polyneuropathy in patients with type 2 diabetes mellitus: A retrospective study

**DOI:** 10.1097/MD.0000000000049467

**Published:** 2026-07-10

**Authors:** Ling Yang, Rensheng Deng, Liling Chen

**Affiliations:** a Endocrinology department, The Lichuan Ethnic Hospital of Traditional Chinese Medicine, Lichuan, Hubei, China; b Endocrinology department, Enshi Tujia and Miao Autonomous Prefecture Central Hospital, Enshi Tujia and Miao Autonomous Prefecture, Hubei, China; c Endocrinology department, Xiantao First People’s Hospital, Xiantao, Hubei, China.

**Keywords:** distal symmetric polyneuropathy, homocysteine, logistic regression, microvascular complications, nerve conduction studies, risk factors, Type 2 diabetes mellitus, vitamin B12

## Abstract

The pathophysiology of distal symmetric polyneuropathy (DSPN) is multifactorial, involving chronic hyperglycemia, oxidative stress, accumulation of advanced glycation end products, impaired microvascular perfusion, metabolic toxicity, and nutritional deficiencies. Although previous studies have identified various risk factors, substantial heterogeneity exists across regions and populations, and most investigations have not comprehensively integrated metabolic markers, microvascular damage, and electrophysiological data. Therefore, a multidimensional assessment of DSPN risk factors is essential to improve early screening and risk-prediction strategies. A total of 120 patients with type 2 diabetes mellitus (T2DM) were enrolled, including 60 with DSPN and 60 without. Compared with the non-DSPN group, patients with DSPN were older, had longer diabetes duration, and demonstrated a higher prevalence of smoking, diabetic retinopathy (DR), and diabetic kidney disease. DSPN patients exhibited poorer glycemic control (higher fasting blood glucose, 2-hour postprandial blood glucose, and hemoglobin A1c [HbA1c]), more pronounced dyslipidemia (elevated triglycerides, reduced high-density lipoprotein cholesterol), and abnormalities in inflammatory and nutritional markers (elevated C-reactive protein and homocysteine [HCY], reduced vitamin B12). Renal function parameters (serum creatinine, estimated glomerular filtration rate, urine albumin–creatinine ratio) indicated more severe microvascular impairment in the DSPN group. Electrophysiological testing showed reduced sensory and motor nerve conduction velocities, decreased amplitudes, and prolonged latencies. Multivariable logistic regression identified diabetes duration, DR, elevated HbA1c, elevated HCY, and reduced sural nerve amplitude as independent risk factors for DSPN. Longer diabetes duration, DR, elevated HbA1c, elevated HCY levels, and reduced sural nerve amplitude were independently associated with DSPN in patients with T2DM. These findings may contribute to improved risk stratification and support further investigation of multidimensional approaches for early DSPN identification. This single-center retrospective cohort study included patients with T2DM treated at our hospital between June 2023 and June 2025. DSPN was diagnosed based on American Diabetes Association, European Federation of Neurological Societies, and diabetic neuropathy working group criteria using a combination of clinical symptoms, neurological signs, and nerve conduction studies. Demographic characteristics, lifestyle factors, diabetes-related complications, glucose and lipid metabolism indicators, renal function, inflammatory and nutritional markers, and electrophysiological parameters were collected. Group differences were assessed using the *t*-test, Mann–Whitney *U* test, or *χ*^2^ test. Variables with *P* < .10 in univariate analyses were entered into multivariable logistic regression to identify independent risk factors for DSPN. Sensitivity analyses and collinearity diagnostics were performed to assess the robustness of the findings.

## 1. Introduction

Type 2 diabetes mellitus (T2DM) is one of the most prevalent chronic metabolic disorders worldwide, and its growing burden has been accompanied by a rising incidence of diabetic peripheral neuropathy, a leading cause of disability among affected individuals.^[1]^ Large international cohort studies indicate that approximately 30 to 50% of patients with T2DM eventually develop distal symmetric polyneuropathy (DSPN), with risk increasing substantially as disease duration lengthens and metabolic disturbances accumulate.^[2,3]^ DSPN typically manifests as distal symmetrical sensory abnormalities, pain, and impaired vibration perception, and represents a major contributor to diabetic foot ulceration, infection, and amputation, as well as to reduced quality of life.^[4,5]^

The pathophysiology of DSPN is multifactorial, involving intertwined metabolic and vascular processes. Chronic hyperglycemia drives oxidative stress, accumulation of advanced glycation end products (AGEs), mitochondrial dysfunction, axonal degeneration, and myelin injury, all of which accelerate structural nerve damage.^[6]^ These metabolic insults, together with reduced microvascular perfusion, ischemia, and chronic low-grade inflammation, act synergistically to promote DSPN onset and progression.^[7,8]^ Landmark trials such as the Diabetes Control and Complications Trial/Epidemiology of Diabetes Interventions and Complications have demonstrated a clear dose-response relationship between hemoglobin A1c (HbA1c) levels and neuropathy incidence, underscoring the central role of long-term glycemic control.^[9]^

In addition to hyperglycemia, a growing body of evidence shows that aging, prolonged diabetes duration, dyslipidemia, hypertension, obesity, and smoking independently increase the likelihood of DSPN.^[10,11]^ Clinical studies have further supported the concept of “microvascular–neural co-pathogenesis,” whereby diabetic retinopathy (DR) and diabetic kidney disease (DKD) exhibit strong associations with DSPN, suggesting shared mechanisms involving inflammation, impaired microcirculation, and neural ischemia.^[12,13]^ Nutritional and metabolic abnormalities (particularly vitamin B12 deficiency and elevated homocysteine [HCY]) have also been implicated in impaired nerve conduction and symptom worsening.^[14]^

Diagnosis of DSPN requires an integrated evaluation of symptoms, neurological signs, and nerve conduction studies (NCS). However, atypical or absent symptoms in early or subclinical stages frequently lead to underdiagnosis. Identifying quantifiable risk factors and developing accurate prediction models has therefore become a key research priority internationally.^[15]^

Although existing studies have explored DSPN mechanisms and risk profiles, substantial heterogeneity remains across regions and populations. Moreover, many investigations have been limited by small sample sizes, incomplete variable collection, or the absence of electrophysiological data, constraining the generalizability of their findings. Real-world, multidimensional analyses are thus needed to more precisely characterize DSPN risk in diverse clinical settings.

Against this backdrop, the present study conducted a retrospective cohort analysis of T2DM patients at our institution. By systematically integrating demographic characteristics, metabolic parameters, microvascular complications, inflammatory and nutritional markers, and electrophysiological data, this study aims to delineate key determinants of DSPN and provide region-specific evidence to support early screening and clinical management.

## 2. Methods

### 2.1. Study design

This study was approved by the Ethics Committee of Xiantao First People’s Hospital. This single-center retrospective cohort study examined risk factors for DSPN in patients with T2DM who attended our hospital between June 2023 and June 2025. Eligible cases were identified from endocrinology outpatient and inpatient records through automated extraction from the electronic health record system, followed by manual verification. The workflow included initial case screening, eligibility assessment, confirmation of neuropathy status, data extraction, handling of missing values, cross-checking, and final statistical analysis, ensuring methodological rigor and data reliability. DSPN diagnoses were independently reviewed by 2 endocrinologists with at least 5 years of experience in diabetic complications. Discrepancies were adjudicated by a senior physician. All data were collected according to a predefined standardized extraction protocol to minimize bias. Diagnostic information was verified using International Classification of Diseases codes, electrophysiology reports, and imaging data. To reduce selection bias, all patients meeting inclusion criteria were consecutively enrolled without sampling. The study was designed and reported in accordance with Strengthening the Reporting of Observational Studies in Epidemiology guidelines. Ethical approval was obtained from the institutional review board, which waived the requirement for informed consent due to the retrospective design. All patient data were anonymized and processed according to the Declaration of Helsinki and privacy protection standards.

### 2.2. Study population

#### 2.2.1. Inclusion criteria

Patients were included if they met the following conditions:

1. a documented diagnosis of T2DM;
2. age between 18 and 85 years;
3. complete clinical records of neuropathic symptoms, neurological examination findings, and at least 1 peripheral nerve conduction study;
4. complete and traceable medical documentation within the study period;
5. adherence to routine follow-up and testing procedures without major data loss.

#### 2.2.2. Exclusion criteria

To minimize confounding, the following were excluded:

1. neuropathies secondary to nondiabetic causes (e.g., chemotherapy, alcohol abuse, toxin exposure, immune-mediated or hereditary neuropathies, vitamin-deficiency neuropathy);
2. acute metabolic decompensation (e.g., ketoacidosis, hyperosmolar crisis);
3. severe organ failure, active malignancy, or severe infection;
4. gestational diabetes or other specific types of diabetes;
5. critical variables missing > 20% or insufficient data to classify neuropathy status.

#### 2.2.3. Diagnostic criteria for DSPN

DSPN was diagnosed according to American Diabetes Association, European Federation of Neurological Societies, and diabetic neuropathy working group recommendations, integrating:

1. typical sensory symptoms (distal numbness, paresthesia, burning pain, or sensory loss);
2. neurological signs (reduced/absent ankle reflexes, impaired vibration, pain, or temperature sensation);
3. electrophysiological abnormalities in at least 2 peripheral nerves (reduced conduction velocity, decreased amplitude, or prolonged latency).

Patients meeting “symptoms/signs + electrophysiological abnormalities” were classified as having DSPN. Individuals with isolated neurophysiological abnormalities but no typical symptoms were considered subclinical and were not included in the DSPN group.

### 2.3. Data collection

Two trained investigators extracted data based on a unified data dictionary, with quality audits by a third researcher. Collected variables included demographics, disease course, lifestyle behaviors, treatment history, chronic complications, laboratory indicators, and electrophysiological parameters. Demographic and clinical information included age, sex, body mass index, diabetes duration, smoking and alcohol consumption, physical activity, sleep quality, blood pressure readings, and hypertension status. Chronic complications recorded included DR, DKD, coronary artery disease, cerebrovascular disease, peripheral arterial disease, and nonalcoholic fatty liver disease. Laboratory variables included fasting glucose, 2-hour postprandial glucose, HbA1c, lipid profile (total cholesterol, triglycerides, low-density lipoprotein cholesterol, high-density lipoprotein cholesterol [HDLC]), renal function (serum creatinine, estimated glomerular filtration rate [eGFR]), urine albumin–creatinine ratio, liver function tests, HCY, vitamin B12, folate, C-reactive protein (CRP), and white blood cell count. Medication use was recorded for insulin, metformin, sodium-glucose cotransporter-2 inhibitors, dipeptidyl peptidase-4 inhibitors, glucagon-like peptide-1 receptor agonists, statins, angiotensin-converting enzyme inhibitor/angiotensin receptor blockers, and antiplatelet therapies. Electrophysiological data extracted included conduction velocity, amplitude, and latency of the common peroneal, tibial, and sural nerves. If available, neuropathy assessment scores (Michigan neuropathy screening instrument, neuropathy symptom score/neuropathy disability score) or quantitative vibration threshold measurements were included.

### 2.4. Statistical analysis

Statistical analyses were conducted using Statistical Package for the Social Sciences version 22.0. Continuous variables were expressed as mean ± standard deviation or median (interquartile range), depending on distribution, and categorical variables as frequencies (%). Group comparisons were performed using independent-sample *t*-tests, Mann–Whitney *U* tests, *χ*^2^ tests, or Fisher exact tests. Variables with *P* < .10 in univariate analyses were entered into multivariable logistic regression to identify independent predictors of DSPN; adjusted odds ratios (OR) and 95% confidence intervals were reported. Sensitivity analyses were performed by stratifying participants according to diabetes duration (< 10 vs ≥ 10 years), glycemic control (HbA1c < 8% vs ≥ 8%), and long-term metformin use. Collinearity was assessed using the variance inflation factor to ensure model stability. All statistical tests were 2-tailed, and significance was set at *P* < .05.

## 3. Results

### 3.1. Baseline demographic and clinical characteristics

A total of 120 patients with T2DM were included, with 60 classified into the DSPN group and 60 into the non-DSPN group. As shown in Table 1, significant between-group differences were observed in several baseline variables. Patients with DSPN were older (64.8 ± 10.9 vs 58.3 ± 11.2 years; *t* = 3.26, *P* = .001), suggesting an age-related increase in neuropathy risk. Diabetes duration was markedly longer in the DSPN group (11.8 ± 6.4 vs 7.6 ± 4.9 years; *t* = 4.09, *P* < .001), consistent with the cumulative nature of diabetic nerve injury. Lifestyle characteristics also differed: the prevalence of smoking was higher in the DSPN group (36.7% vs 20.0%; *χ*^2^ = 4.24, *P* = .039), indicating that tobacco-related vascular impairment may contribute to neuropathy. Chronic complications were more common among DSPN patients, including DR (46.7% vs 20.0%; *P* = .001) and DKD (31.7% vs 15.0%; *P* = .026), supporting the shared microvascular pathology underlying these conditions. Systolic blood pressure was also significantly elevated in the DSPN group (141.6 ± 18.7 vs 134.4 ± 16.9 mm Hg; *P* = .030). Collectively, age, diabetes duration, smoking, hypertension, DR, and DKD were all associated with DSPN occurrence.

**Table 1:** Comparison of baseline demographic and clinical characteristics between groups.

Variable	DSPN Group (n = 60)	Non-DSPN Group (n = 60)	*t*/*χ*^2^	*P* value
Age (yrs)	64.8 ± 10.9	58.3 ± 11.2	3.26	.001
Male (n [%])	38 (63.3)	32 (53.3)	1.17	.279
BMI (kg/m^2^)	24.1 ± 3.6	23.2 ± 3.1	1.42	.158
Diabetes duration (yrs)	11.8 ± 6.4	7.6 ± 4.9	4.09	< .001
Smoking history (n [%])	22 (36.7)	12 (20.0)	4.24	.039
Alcohol use (n [%])	18 (30.0)	11 (18.3)	2.12	.145
Hypertension (n [%])	34 (56.7)	23 (38.3)	4.41	.036
Coronary artery disease (n [%])	16 (26.7)	8 (13.3)	3.35	.067
DR (n [%])	28 (46.7)	12 (20.0)	10.33	.001
DKD (n [%])	19 (31.7)	9 (15.0)	4.94	.026
SBP (mm Hg)	141.6 ± 18.7	134.4 ± 16.9	2.2	.03
DBP (mm Hg)	82.3 ± 11.5	79.7 ± 10.6	1.24	.218

BMI = body mass index, DBP = diastolic blood pressure, DR = diabetic retinopathy, DKD = diabetic kidney disease, DSPN = distal symmetric polyneuropathy, n= number of participants, SBP = systolic blood pressure.

### 3.2. Glycemic control

As summarized in Table 2, glycemic control was significantly poorer in patients with DSPN. Fasting glucose levels were higher (9.6 ± 2.3 vs 8.4 ± 2.1 mmol/L; *t* = 2.92, *P* = .004), as were postprandial glucose levels (2-hour postprandial blood glucose: 15.3 ± 4.2 vs 13.1 ± 3.8 mmol/L; *t* = 3.05, *P* = .003). HbA1c was substantially elevated in the DSPN group (9.1 ± 1.5% vs 8.0 ± 1.2%; *t* = 4.15, *P* < .001), reinforcing the central role of long-term hyperglycemia in nerve damage. These findings indicate that both short-term glucose fluctuations and chronic glycemic burden may promote oxidative stress and impair microvascular perfusion, accelerating neural injury.

**Table 2:** Comparison of glycemic parameters between groups.

Variable	DSPN Group	Non-DSPN Group	*t*	*P* value
FBG (mmol/L)	9.6 ± 2.3	8.4 ± 2.1	2.92	.004
2h-PBG (mmol/L)	15.3 ± 4.2	13.1 ± 3.8	3.05	.003
HbA1c (%)	9.1 ± 1.5	8.0 ± 1.2	4.15	< .001

2h-PBG = 2-hour postprandial blood glucose, DSPN = distal symmetric polyneuropathy, FBG = fasting blood glucose, HbA1c = hemoglobin A1c.

### 3.3. Lipid profile, nutritional markers, and inflammatory indicators

Table 3 shows significant differences in lipid metabolism and nutritional parameters. Triglyceride levels were higher in the DSPN group (2.01 vs 1.67 mmol/L; *P* = .038), whereas HDLC levels were lower (1.00 vs 1.15 mmol/L; *P* = .035). These abnormalities may compromise neural microcirculation and promote axonal degeneration. Marked variations were also observed in nutritional and metabolic markers. Vitamin B12 concentrations were significantly reduced in DSPN patients (278 vs 334 pg/mL; *P* = .004), while HCY levels were elevated (17.8 vs 14.9 μmol/L; *P* = .001). Both indicators are closely linked to myelin integrity and axonal metabolism. CRP levels were higher in the DSPN group (5.3 vs 4.1 mg/L; *P* < .001), supporting a role for chronic inflammation in neuropathogenesis. Overall, these results suggest that dyslipidemia, micronutrient imbalance, and low-grade inflammation may interact to exacerbate nerve injury.

**Table 3:** Comparison of lipid profile, nutritional markers, and inflammatory indicators.

Variable	DSPN Group	Non-DSPN Group	*t*	*P* value
TC (mmol/L)	5.02 ± 1.1	4.78 ± 1.0	1.16	.25
TG (mmol/L)	2.01 ± 0.9	1.67 ± 0.8	2.1	.038
LDLC (mmol/L)	3.02 ± 0.8	2.76 ± 0.7	1.84	.069
HDLC (mmol/L)	1.00 ± 0.3	1.15 ± 0.4	2.14	.035
Vitamin B12 (pg/mL)	278 ± 91	334 ± 106	2.98	.004
HCY (μmol/L)	17.8 ± 4.7	14.9 ± 4.3	3.44	.001
CRP (mg/L)	5.3 ± 1.8	4.1 ± 1.5	3.82	< .001

CRP = C-reactive protein, DSPN = distal symmetric polyneuropathy, HCY = homocysteine, HDLC = high-density lipoprotein cholesterol, LDLC = low-density lipoprotein cholesterol, TC = total cholesterol, TG = triglycerides.

### 3.4. Renal function and albuminuria

As shown in Table 4, renal function deteriorated more markedly in the DSPN group. Serum creatinine levels were higher (87.3 vs 78.4 μmol/L; *P* = .026), while eGFR was significantly reduced (78.5 vs 90.8 mL/min/1.73m^2^; *P* = .001). urine albumin–creatinine ratio, a sensitive indicator of microvascular impairment, was markedly increased (59.4 vs 32.1 mg/g; *P* < .001). These findings reinforce the concept of “microvascular–neuropathy co-morbidity,” highlighting the shared vascular component contributing to neural dysfunction.

**Table 4:** Comparison of renal function and albuminuria.

Variable	DSPN Group	Non-DSPN Group	Statistic	*P* value
Scr (μmol/L)	87.3 ± 22.1	78.4 ± 19.6	*t* = 2.26	.026
eGFR (mL/min/1.73m^2^)	78.5 ± 18.7	90.8 ± 20.2	*t* = 3.25	.001
UACR (mg/g)	59.4 (33–104)	32.1 (15–63)	*Z* = 3.92	< .001

DSPN = distal symmetric polyneuropathy, eGFR = estimated glomerular filtration rate, Scr =serum creatinine, UACR = urine albumin–creatinine ratio.

### 3.5. Medication use

Medication profiles are summarized in Table 5. Insulin use was more frequent in the DSPN group (63.3% vs 43.3%; *P* = .027), likely reflecting longer disease duration and more challenging glycemic control. Use of metformin, statins, and other antihyperglycemic agents did not differ significantly; however, these therapies may indirectly influence neuropathy risk through effects on metabolic control. Long-term metformin exposure, for instance, may contribute to B12 deficiency. Although most differences were not statistically significant, medication patterns may serve as indicators of disease severity and metabolic stability.

**Table 5:** Comparison of medication use.

Medication	DSPN Group n (%)	Non-DSPN Group n (%)	*χ* ^2^	*P* value
Metformin	46 (76.7)	49 (81.7)	0.46	.499
Insulin	38 (63.3)	26 (43.3)	4.88	.027
SGLT2 inhibitors	12 (20.0)	18 (30.0)	1.36	.244
DPP-4 inhibitors	20 (33.3)	28 (46.7)	2.35	.125
Statins	42 (70.0)	36 (60.0)	1.34	.247

DPP-4 = dipeptidyl peptidase-4, DSPN = distal symmetric polyneuropathy, n= number of participants, SGLT2 = sodium-glucose cotransporter-2.

### 3.6. Nerve conduction study findings

Electrophysiological parameters differed markedly between groups (Table 6). Sensory nerve conduction velocity of the sural nerve was significantly reduced in DSPN patients (35.2 vs 41.6 m/s; *P* < .001), with a pronounced decline in amplitude (4.5 vs 7.2 μV; *P* < .001). Motor conduction velocity of the tibial nerve was also decreased (37.6 vs 42.9 m/s; *P* < .001), accompanied by prolonged latency (5.1 vs 4.4 ms; *P* < 001). These results reflect characteristic features of axonal degeneration and demyelination. Electrophysiological abnormalities provide objective quantification of neuropathy severity and correlate with clinical manifestations and long-term prognosis.

**Table 6:** Nerve conduction study findings.

Variable	DSPN Group	Non-DSPN Group	*t*	*P* value
Sural sensory conduction velocity (m/s)	35.2 ± 4.3	41.6 ± 3.9	8.27	< .001
Sural amplitude (μV)	4.5 ± 1.8	7.2 ± 2.1	7.14	< .001
Tibial motor conduction velocity (m/s)	37.6 ± 5.1	42.9 ± 4.8	5.27	< .001
Tibial latency (ms)	5.1 ± 0.7	4.4 ± 0.6	5.57	< .001

DSPN = distal symmetric polyneuropathy.

### 3.7. Univariate analysis

Univariate logistic regression (Table 7) identified several variables significantly associated with DSPN, including age (OR = 1.05, *P* = .002), diabetes duration (OR = 1.12, *P* < .001), DR (OR = 3.46, *P* = .002), DKD (OR = 2.57, *P* = .029), HbA1c (OR = 1.68, *P* < .001), HCY (OR = 1.12, *P* = .001), and vitamin B12 (OR = 0.995, *P* = .013). These predictors span glycemic burden, microvascular injury, metabolic disturbances, and nutritional deficiencies, underscoring DSPN as a multifactorial condition.

**Table 7:** Univariate logistic regression analysis.

Variable	OR	95% CI	*P* value
Age (yrs)	1.05	1.02–1.09	.002
Diabetes duration (yrs)	1.12	1.06–1.19	< .001
DR	3.46	1.57–7.62	.002
DKD	2.57	1.10–6.02	.029
HbA1c (%)	1.68	1.28–2.31	< .001
HCY	1.12	1.05–1.20	.001
Vitamin B12	0.995	0.991–0.999	.013

CI = confidence interval, DKD = diabetic kidney disease, DR = diabetic retinopathy, HbA1c = hemoglobin A1c, HCY = homocysteine, OR = odds ratio.

### 3.8. Multivariate analysis

In the multivariable logistic regression model (Table 8), several variables remained independently associated with DSPN: diabetes duration (OR = 1.09, *P* = .010), DR (OR = 2.72, *P* = .030), HbA1c (OR = 1.41, *P* = .022), HCY (OR = 1.08, *P* = .027), and sural nerve amplitude (OR = 0.71, *P* = .003). These findings highlight the contributions of chronic hyperglycemia, microvascular compromise, metabolic toxicity, and electrophysiological impairment to DSPN pathogenesis. The inverse association of sural amplitude with DSPN suggests that neurophysiological alterations may not only reflect existing damage but may also serve as an early predictor of disease risk.

**Table 8:** Multivariate logistic regression analysis.

Variable	OR	95% CI	*P* value
Diabetes duration (yrs)	1.09	1.02–1.17	.01
DR	2.72	1.10–6.76	.03
HbA1c (%)	1.41	1.05–1.90	.022
HCY	1.08	1.01–1.16	.027
Sural nerve amplitude	0.71	0.57–0.89	.003

CI = confidence interval, DR = diabetic retinopathy, HbA1c = hemoglobin A1c, HCY = homocysteine, OR = odds ratio.

## 4. Discussion

In this retrospective study of patients with T2DM, we identified diabetes duration, DR, HbA1c, HCY, and sural nerve amplitude as independent factors associated with DSPN. A notable aspect of the present study is the simultaneous evaluation of metabolic, microvascular, nutritional, inflammatory, and electrophysiological variables within a single analytical framework. These findings support the multifactorial nature of DSPN and suggest that integrating clinical and neurophysiological indicators may improve risk assessment beyond traditional metabolic measures alone.

Consistent with prior evidence, patients with DSPN were older and had a substantially longer duration of diabetes, emphasizing the cumulative burden of metabolic and vascular injury over time. Aging is known to impair peripheral nerve regeneration, reduce neurotrophic support, and diminish antioxidant capacity,^[16]^ rendering nerves more susceptible to metabolic toxicity. Prolonged exposure to hyperglycemia, AGEs, oxidative radicals, and chronic ischemia contributes to progressive myelin disruption and axonal degeneration.^[17]^ These mechanisms align with the associations identified in our cohort and underscore the need for intensified neuropathy screening in older individuals and those with long-standing diabetes.

Glycemic indices (including fasting blood glucose, 2-hour postprandial blood glucose, and HbA1c) were significantly elevated in the DSPN group, reaffirming the central role of glycemic burden in neuropathy. Findings from the Diabetes Control and Complications Trial/Epidemiology of Diabetes Interventions and Complications trial demonstrated a clear dose-response relationship between glycemic levels and neural injury.^[18]^ Hyperglycemia induces oxidative stress, activates the polyol pathway, promotes AGEs cross-linking, and drives inflammatory signaling, all of which contribute to structural nerve damage.^[19]^ Moreover, glycemic variability has been shown to induce oxidative surges and endothelial dysfunction more profoundly than chronic hyperglycemia alone.^[20]^ These insights highlight the importance of managing both average glucose and fluctuations in clinical practice.

Abnormal lipid metabolism also emerged as a relevant factor. Higher triglycerides and lower HDLC in the DSPN group support the notion that dyslipidemia impairs endothelial function, reduces neural blood flow, and enhances lipotoxicity.^[21]^ The elevated CRP and HCY concentrations further illustrate the combined influence of inflammation and metabolic toxicity. CRP has been associated with impaired nerve conduction and structural fiber damage,^[22]^ while HCY contributes to demyelination, oxidative stress, and DNA methylation disturbances.^[23]^ Lower vitamin B12 levels in DSPN patients suggest additional vulnerability to myelin injury and slowed conduction velocity.^[24]^ Collectively, these findings support a “metabolic–inflammatory–nutritional axis” contributing to neuropathy and highlight the need for broader metabolic profiling during routine evaluations.

Microvascular disease was significantly more common in DSPN patients. DR remained an independent predictor after multivariable adjustment, underscoring the shared microangiopathic mechanisms linking these complications. Endothelial dysfunction, basement membrane thickening, and impaired capillary perfusion promote chronic neural ischemia and increase susceptibility to metabolic damage.^[25]^ DKD may also exacerbate neuropathy via albuminuria, reduced eGFR, and heightened systemic inflammation, with multiple studies showing strong coupling between DKD and DSPN.^[26]^ Thus, microvascular complications serve not only as comorbidities but also as clinical indicators of rising neuropathy risk.

Electrophysiological abnormalities were pronounced in DSPN patients. Reduced sural nerve amplitude, slowed conduction velocity, and prolonged latency reflect the dual processes of axonal loss and demyelination. Prior studies identified reduced sural amplitude as an early marker of axonal degeneration,^[27]^ and decreasing NCS parameters may precede clinical symptoms by several years.^[28]^ Moreover, NCS abnormalities have been validated as reliable prognostic biomarkers for neuropathy progression.^[29]^ In this study, sural amplitude remained an independent factor in multivariable analysis, suggesting that neurophysiological testing should be incorporated into routine evaluation to identify high-risk patients at early stages.

Although medication use did not differ significantly between groups, higher insulin usage among DSPN patients may reflect poorer metabolic control and longer disease duration. Additionally, long-term metformin therapy has been associated with vitamin B12 deficiency, potentially contributing to neuropathy in susceptible patients.^[23]^ Medication patterns should therefore be interpreted not only as therapeutic choices but also as indirect indicators of disease severity and metabolic balance.

Overall, DSPN appears to arise from the interplay of chronic hyperglycemia, dyslipidemia, inflammation, micronutrient imbalance, microvascular dysfunction, and electrophysiological impairment. These findings align with recently published evidence from China emphasizing multidimensional mechanisms underlying diabetic neuropathy.^[30]^ Our findings support closer neurological surveillance in patients with established risk factors such as longer diabetes duration, poor glycemic control, and microvascular complications. In addition, elevated HCY levels and lower vitamin B12 concentrations were associated with DSPN in this cohort. Although these markers are not currently recommended as routine screening targets in major guidelines, they may warrant further investigation as potential components of future risk-stratification strategies.

Such strategies may delay neuropathy progression and reduce the burden of serious complications such as diabetic foot. Strengths of this study include the incorporation of electrophysiological data, strict inclusion/exclusion criteria, and sensitivity analyses enhancing robustness. Limitations include the single-center retrospective design, missing data for certain clinical scores (e.g., Michigan neuropathy screening instrument, neuropathy disability score), and lack of continuous glucose monitoring metrics, which may better reflect glycemic variability. Future studies with prospective designs and larger cohorts are needed to validate predictive models and evaluate modifiable risk factors.

## Author contributions

**Conceptualization:** Ling Yang, Rensheng Deng, Liling Chen.

**Data curation:** Ling Yang, Rensheng Deng, Liling Chen.

**Formal analysis:** Ling Yang, Rensheng Deng, Liling Chen.

**Funding acquisition:** Ling Yang, Liling Chen.

**Investigation:** Liling Chen.

**Writing** – **original draft:** Liling Chen.

**Writing** – **review & editing:** Liling Chen.
